# A Taxonomy Proposal for the Assessment of the Changes in Soundscape Resulting from the COVID-19 Lockdown

**DOI:** 10.3390/ijerph17124205

**Published:** 2020-06-12

**Authors:** César Asensio, Pierre Aumond, Arnaud Can, Luis Gascó, Peter Lercher, Jean-Marc Wunderli, Catherine Lavandier, Guillermo de Arcas, Carlos Ribeiro, Patricio Muñoz, Gaetano Licitra

**Affiliations:** 1 Instrumentation and Applied Acoustics Research group (I2A2), Universidad Politécnica de Madrid, 28031 Madrid, Spain; luisgascosanchez@gmail.com (L.G.); g.dearcas@upm.es (G.d.A.); 2 UMRAE, Univ Gustave Eiffel, IFSTTAR, CEREMA, 44340 Bouguenais, France; pierre.aumond@univ-eiffel.fr (P.A.); arnaud.can@ifsttar.fr (A.C.); 3 Institute for Highway Engineering and Transport Planning, Graz University of Technology, 8010 Graz, Austria; peter.lercher.at@gmail.com; 4 Empa, Swiss Federal Laboratories for Material Science and Technology, Laboratory for Acoustics/Noise Control, 8600 Dübendorf, Switzerland; jean-marc.wunderli@empa.ch; 5 ETIS Laboratory, UMR 8051, CY Cergy Paris University, ENSEA, CNRS, F-95302 Cergy-Pontoise Cedex, France; catherine.lavandier@cyu.fr; 6 Bruitparif, 93200 Saint-Denis, France; Carlos.Ribeiro@bruitparif.fr; 7 Acoucite, Observatoire de l’environnement sonore de la Métropole de Lyon, 69007 Lyon, France; patricio.munoz@acoucite.org; 8 Environmental Protection Agency of Tuscany Region, Pisa Department, 56127 Pisa, Italy

**Keywords:** COVID-19, noise, soundscape, metrics, indicators, descriptors, sound, lockdown

## Abstract

Many countries around the world have chosen lockdown and restrictions on people’s mobility as the main strategies to combat the COVID-19 pandemic. These actions have significantly affected environmental noise and modified urban soundscapes, opening up an unprecedented opportunity for research in the field. In order to enable these investigations to be carried out in a more harmonized and consistent manner, this paper makes a proposal for a set of indicators that will enable to address the challenge from a number of different approaches. It proposes a minimum set of basic energetic indicators, and the taxonomy that will allow their communication and reporting. In addition, an extended set of descriptors is outlined which better enables the application of more novel approaches to the evaluation of the effect of this new soundscape on people’s subjective perception.

## 1. Introduction

Unfortunately, the year 2020 will be known as the year of the coronavirus disease (COVID-19) pandemic. To a greater or lesser extent, the epidemic has spread to every continent, without distinction, affects all ages and is particularly dangerous for older people. The strategies designed by different governments to combat the pandemic in many countries have been very diverse, but many countries have chosen lockdown and restrictions on people’s mobility [1]. More than 3.9 billion people, or a half of the world’s population living in 90 different countries around the world have been under containment as a measure to maintain social distancing [2].

Commercial flights, both international and domestic, have been severely restricted, with all flights not dedicated to the provision of medical supplies and other essential products being affected in many countries [3]. Likewise, ground transportation has also been severely restricted, with substantial percentages of the population unable to access their jobs or having to work remotely [4].

In addition to the dreadful consequences that the pandemic has had on the population, in terms of infections, hospitalizations and the number of deaths, the lockdown of people and their absence from the environment has had considerable environmental consequences, with animal species returning to the urban environment and beaches, and varying reductions in peak and average air pollution levels in populated areas [5,6,7].

As a result of restrictions on urban mobility, traffic noise has been drastically reduced. Conversely, natural noise, such as bird singing, is emerging again, although it is difficult to know whether this is related to a closer presence of the source, an increase in levels, lack of masking noise or a perceptual effect, and whether it is due to the lockdown or not [8].

Therefore, the acoustics community has been mobilized. National acoustical associations in Italy and UK launched initiatives to collect measurement campaign data [9,10] and many consultants, engineers, research groups and noise management authorities around the world have begun to produce reports to address, through measurement data, the assessment of the reduction that confinement has produced in the environmental noise of each city. Although a few of the initiatives gave some general indications, there is a risk that these interesting reports, coming from personal and structured actions, suffer from a lack of consistency that makes it almost impossible to compare them, which would be extremely challenging for the overall analysis of the effect on the confinement on human behaviors and perception.

At the same time, new projects are active to collect recordings and metadata of sounds in the COVID-19 scenario, such as the LYS (locate your sound) project [11] in Italy with around 3000 recordings on 6 May 2020, showing the richness of lived experiences and the value of the recordings so that people do not forget and recover lost sounds. Also, through sound recording and automatic audio tagging of recordings, the Silent-Cities Project aims to create a database of audio files that allows to study, among other things, the relationship between natural and human-generated sounds in different levels of economic activity [12]. Also related to this topic, Acoucité has developed a questionnaire oriented towards assessing population feelings about the changes in the noise environment since lockdown [13].

Since it is expected that in the coming months these preliminary analyses will become scientific articles, it is considered very necessary to establish a common framework to harmonize the basic results of these investigations, so that comparisons can be made between different populations and countries, leading to a macro-analysis that will make it possible to know and evaluate the overall effect of confinement, to compare the effect of different confinement strategies and to communicate this information to the public.

In order to achieve these objectives, in this communication we propose a minimum set of common descriptors, which will make it possible to assess noise pollution in each location, and to appraise the noise reduction that the measures against COVID-19 imply. In addition, to give a status of open data to all this information, and to facilitate future analyses, we propose a data structure that gathers all the noise-related data information in the form of a taxonomy. Although this data structure arises as a necessity for the comparison of noise studies related to COVID-19 effects, it should also be valid for the assessment of noise in the future, with minor changes both in exceptional and everyday circumstances.

## 2. Noise Descriptors and Taxonomy for Physical Characterization

This paper focuses on indicators for physical characterization of noise, since an important part of the analysis will probably deal with the pre-post comparison based on the noise monitoring systems implemented in cities and airports. Indicators that aim to assess people’s exposure to noise are widespread [14]. With their benefits and shortcomings, they allow a description to be made based on objective criteria, such as the acoustic energy contained in the environment.

### 2.1. Measurement Data Structure

We recommend that each measurement be described by the following set of data, which will refer to a time interval starting at the day and time referenced. We propose to use a simple open file format such as the comma-separated values (CSV) file to share the raw data. The field names of the first row of the dataset are shown in Table 1, and each row of the file will describe a measurement.

It is recommended that in each location, the basic data set reported on a daily basis be L_n_ and L_den_, following the recommendations of the Environmental Noise Directive [15]. Additionally, it is considered convenient to add, if available, as an extended data set, the time series of measurements of equivalent sound level of one hour (either A or Z weighted, L_Aeq,1h,_ L_eq,1h_). Therefore, this recommendation includes 24 descriptors a day (24 L_Aq,1 h_ or L_eq,1 h_ values). The same data structure can be valid for daily or hourly basis, using the time of indicator definition, duration and starting time.

It is necessary to ensure the reliability of the data, so that measurements that could be affected by weather, maintenance operations or unusual sound events, that could affect the measurements, are excluded.

Table S1, provided as supplementary material, contains an example of a data file, according to this measurement data structure.

### 2.2. Data to Report

For data processing and reporting, local diversities and uses may result in large differences that prevent comparison of results. Each study can have a very different scope and objectives, and thus the results reported can vary considerably. However, we consider that analyzing the reduction of noise produced during lockdown may be an objective common to all of them, and, focusing on the evaluation of such reduction, we propose a series of indicators that may be useful, considering them as a set of minimums that all studies should address. For this reason, we recommend that the reports contain, at least, a time series (chart or table) for L_den_ and L_n_, and the information specified in Table 2:

In some locations, due to their characteristics, it may be of interest to evaluate the reduction occurring during weekends or holidays. In this case, the information contained in Table 2 can be replicated, redefining the peak and valley hours, depending on the prevailing noise source in each area.

### 2.3. Data Collection

Although data collection is out of the scope of this communication, we encourage providers to share their database with the community on the Zenodo platform, which is an open-access repository operated by CERN [17]. For each submission, a persistent digital object identifier (DOI) is given, which makes the stored items easily citable. The upload limit is about 50 GB. To identify all the databases that will have followed the protocol recommended in this communication. Please add the tags: “Noise”, “COVID-19”; “Lockdown”, “Taxonomy”.

## 3. Extended Indicators 

The previous section focuses on describing the noise dose, and how it has decreased because of the reduced mobility and human activities that confinement has produced. This is an aspect that has been well studied over decades, so it has been relatively easy to agree on a set of data, which we believe noise monitoring systems will be recording on a regular basis.

However, this set of indicators does not fully describe the subjective experience that the new soundscape draws. Sudden shift in sound environments include changes in noise dynamics, and the emergence of unusual sound sources. Beyond the purely energetic effect that derives from the confinement, it is foreseeable that the perception of change in the soundscape will be different according to cultural aspects [18,19]. This can only be widely investigated if an adequate set of descriptors, conveniently harmonized at international level, are defined. This requires an extended set of indicators needed for more detailed analyses.

These types of investigations are not so widespread in the different areas of noise management in public administrations, and therefore there are restrictions with respect to the technical knowledge of the staff who must carry out the measurements. This is the reason why we wanted to include a classification of indicators in this paper, that may be helpful for future research, and which may still be used to describe outdoor sound in the face of the unique phenomenon we are experiencing, from different points of view, such as biophony or soundscape. 

Table 3 also includes the energetic indicators already mentioned in the previous section, to give consistency, and to allow comparison of the different types of noise descriptors. The following indicators should be calculated on an hourly basis.

## 4. Conclusions

The COVID-19 pandemic has significantly modified urban sound environments, opening up an unprecedented opportunity for research in the field. In order to enable these investigations to be carried out in a more harmonized and consistent manner, the group of experts implied in this article agreed on a minimum set of indicators that should imperatively be calculated. Recommendations are also given as concerning the measurement data structure (taxonomy) for the global assessment of the effect that the lockdown due to COVID-19 has produced on environmental noise.

Beyond this minimum, the selection of a set of descriptors that are capable of adequately describing citizens’ perception of any new circumstance would be highly desirable, to serve as a guide for future research. For this reason, an overview of an extended set of indicators is presented. These indicators cover all the physical dimensions of sound environments, and are supported by elements of literature: Energetic, spectral and temporal dimension, emergence and source-related indicators. Thus, this extended set of indicators should allow a more detailed analysis of the changes in noise environments related to confinement, and to a broader extent help in understanding the impact on sound environments of any policy achieved at the urban scale.

Finally, the COVID-19 crisis has revealed a big lack in the current state-of-the-art to analyze urban sound environments. The noise indicators mainly deal with sound environments as a whole, and do not distinguish between the sound sources that compose it. The sound environments introduced by the lockdowns modified them not only in levels, but also by the present sources. Natural sounds are heard again, both because there is less noise to mask them, and because of the reappearance of animal species in areas usually occupied by vehicles and people. In these circumstances, even the sounds that were previously integrated to form our acoustic environment now, in isolation, acquire a very particular character, and may be especially relevant. When the passage of a vehicle was hidden by the noise of traffic as a whole, now the movement of each vehicle acquires a whole different meaning. Not to mention other sounds, such as the passing of ambulances, which in the pandemic may intensify their meaning and fully change people’s perception.

This dimension is unfortunately absent from current indicators. Therefore, the development of source-orientated indicators, able to quantify the presence of sources of interest, and ideally performing with urban sound mixtures with strong temporal overlaps, is strongly advocated. Premises towards such indicators can be found in the literature, relying on sound recognition [25,47,48,49].

The physical indicators proposed, although they are linked to perceptual and health effects, will most likely be insufficient to capture the entire sound experience. Sensitive data, such as the speed of the experienced change, the link that can exist between the sound environment and its emotional evocation, the diversity in the life situations of city dwellers faced with the lockdown, cannot be captured by physical indicators. They are, however, still an integral part of the soundscapes during this period. Although emphasized in this specific period, this lack stands for any observed modification in sound environments. This advocates for the collection of sensitive data, in addition to physical data, as part of the next generation of measurement networks [49,50].

## Figures and Tables

**Table 1:** Measurement data structure.

Field	Description	Data Type
Identification	Short name, to identify the measurement location	String
City	City	String
Country	Country	String
Measurement provider	Entity that is providing the measurements (i.e., local authority or airport manager)	String
Coordinates	Measurement location, WGS84 format	String latitude, longitude “48.856614; 2.3522219”
Instrument class	Certified instruments should be considered, either type 1 or 2. Non-certified (but calibrated) sensors, type 3	Integer (1, 2, 3)
Instrument brand	Type of area (residential, hospital, school, ...)	String
Prevailing sound sources	Semicolon delimited tags to describe the area, showing the prevailing sound sources	String (road, air, rail, nightlife, etc.)
Date/Time	Measurement starting date and o’clock time	String YYYYmmddThh0000
Stage	Before lockdown = 1 Lockdown = 2 After lockdown = 3	Integer (1, 2, 3)
Description of the stage	A qualitative description of the period to analyze. It will be used to understand the level of lockdown in the city where the measurements were taken. Some tags are proposed.	String. Using tags: (a) events suspended; (b) schools closed; (c) non-essential shops closed; (d) non-essential movement banned; (e) land border closed; (f) non-essential production closed [16]
Duration	Measurement duration. Only necessary for indicator type L_eq_.	Integer (minutes)
Indicator	Type of indicator	String (L_eq_, L_den_, L_n_...)
Frequency weighting	Frequency weighting	String (A, Z)
Measurement	The value of the indicator	Float, 1 decimal digit (decibel)
Miscellaneous	Free comment about the data collection	String

**Table 2:** Minimums to report.

Measurement Location:	Identification	STAGE		
		Before	Lockdown	After
Working day	% days exceeding L_den_ = 65			
	% days exceeding L_n_ = 55			
	Average L_Aeq,1 h_ during rush hour (dBA)			
	Average L_Aeq,1 h_ during off-peak hour (dBA)			
	Average L_den_ (dBA)			
	Average L_n_ (dBA)			

Notes: Arithmetic averages must be considered. The “Before” stage is the one that determines rush and off-peak hour. It will be different for working days and weekends.

**Table 3:** Extended indicators.

	Indicators and Description	Physical Descriptive Power	Perceptive Descriptive Power
Energetic indicators	L_eqT_ continuous equivalent sound pressure level during time period T L_n_ continuous equivalent sound pressure level during night period L_den_, day, evening, night combined indicator [20,21,22]	Cumulative energetic indicators. A, C or Z frequency weighting	Correlated to long term health effects
Statistical indicators	L_90_ [23], 90% percentile level	Describes background noise	Does not emerge from studies
	L_50_, 50% percentile level [24]	Good for discriminating sound environments	Very good correlation with perceived sound intensity and sound pleasantness
	L_10_, 10% percentile level [23,24,25]	Describes contribution of loudest events	Outperforms L_Aeq_ to describe perception of high noise levels
Spectrum and source related indicators	Sound ecology indicators: NDSI, normalized difference soundscape index; ACI, acoustic complexity in; entropy; BIO, bioacoustic index; ADI, acoustic diversity index; AEI, acoustic evenness index [11,26]	Good for discriminating presence of biophonic sounds and anthropogenic sounds in urban sound	Likely to be correlated with the time presence of the described sound sources
	The normalized time and frequency second derivative: TFSD_mean, 4k Hz (birds);_ TFSD_mean,500 Hz (human voices)_ [27,28]	Can be computed from octave band 1 s dataset. Good for discriminating presence of biophonic sounds and anthropogenic sounds in urban sound environment	Likely to be correlated with the time presence of the described sound sources
	L_eq_ (63 Hz–500 Hz); 1/3 octave band continuous sound pressure level [28,29]	Good for discriminating sound environments frequency content	Correlated with the time presence of Traffic
	L_Ceq_-L_Aeq_, difference between A- and C-weighted equivalent continuous sound levels [30,31,32,33,34]	Describes the amount of low frequencies	Differences of 15 to 20 dB show an effect on annoyance and perception of vibrations
Emergences and noise variation indicators	L_Amax_, maximum A-weighted noise level; NA, number of events above a threshold; time above a threshold [35,36]	NA80, number of events above a 80 dBA, or TA80 time above 80 dBA (additional thresholds can be considered)	Awakening probability with increasing L_Amax_ The number of high noise level events may affect sleep motility. For aircraft noise, also an effect on annoyance is suggested
	Calculated from percentiles. Fluctuation: defined as the difference between the (single) source event and the source background level. Emergence: Difference between the source event and the overall background level (L10–L90 or L1–L99) [37,38,39,40,41,42]	Good description of the energetic increase produced by a source	Field investigations on annoyance and hypertension yield some support in the context of mixed sound exposure and low background levels (main roads). No consensus concerning the perceptive effects
	Intermittency ratio (IR). Ratio between the sound energy contributions of events, and the overall contributions during the measurement period [43,44,45,46]	Expresses the energetic share of noise exposure created by individual noise events	Highly intermittent nocturnal noise is correlated with increased risk of cardiovascular diseases. In a fully adjusted hypertension model the IR made an additional contribution beyond the L_den_ in mixed source exposure situations. IR has an additional effect on %HA and can explain shifts of the exposure-response curve of up to about 6 dB.

## Supplementary Materials

The following are available online at https://www.mdpi.com/1660-4601/17/12/4205/s1, Table S1: Data file example.
